# Preclinical Toxicological Characterization of Porphyrin-Doped Conjugated Polymer Nanoparticles for Photodynamic Therapy

**DOI:** 10.3390/pharmaceutics17050593

**Published:** 2025-05-01

**Authors:** Matías Daniel Caverzan, Ana Belén Morales Vasconsuelo, Laura Cerchia, Rodrigo Emiliano Palacios, Carlos Alberto Chesta, Luis Exequiel Ibarra

**Affiliations:** 1 Instituto de Investigaciones en Tecnologías Energéticas y Materiales Avanzados (IITEMA), Universidad Nacional de Río Cuarto (UNRC) y Consejo Nacional de Investigaciones Científicas y Técnicas (CONICET), Río Cuarto X5800BIA, Argentina; dcaverzan@ayv.unrc.edu.ar (M.D.C.); rpalacios@exa.unrc.edu.ar (R.E.P.); cchesta@exa.unrc.edu.ar (C.A.C.); 2 Instituto de Biotecnología Ambiental y Salud (INBIAS), Universidad Nacional de Río Cuarto (UNRC) y Consejo Nacional de Investigaciones Científicas y Técnicas (CONICET), Rio Cuarto X5800BIA, Argentina; amorales@exa.unrc.edu.ar; 3 Institute of Endotypes in Oncology, Metabolism and Immunology “Gaetano Salvatore”, National Research Council, 80131 Naples, Italy; laura.cerchia@cnr.it

**Keywords:** conjugated polymer nanoparticles, biocompatibility, toxicity evaluation, single and repeated dose toxicity, photodynamic therapy

## Abstract

**Background:** Photodynamic therapy (PDT) utilizing nano-based photosensitizers (PSs) offers promising cancer treatment potential but requires rigorous safety evaluation. Conjugated polymer nanoparticles (CPNs) doped with porphyrins, such as platinum porphyrin–doped poly(9,9-dioctylfluorene-alt-benzothiadiazole) (F8BT), exhibit enhanced photodynamic efficiency but lack comprehensive preclinical toxicity data. This study aimed to evaluate the biocompatibility, biodistribution, and acute/subacute toxicity of these CPNs to establish their safety profile for clinical translation. **Methods**: CPNs were synthesized via nanoprecipitation using amphiphilic stabilizers (PSMA or PS-PEG-COOH) and characterized for colloidal stability in parenteral solutions. Hemolysis assays were used to assess blood compatibility. Single-dose (0.3 and 1 mg/kg, intravenous) and repeated-dose (0.1–1 mg/kg, intraperitoneal, every 48 h for 28 days) toxicity studies were conducted in BALB/c mice. Hematological, biochemical, histopathological, and biodistribution analyses (via ICP-MS) were performed to evaluate systemic and organ-specific effects. **Results**: CPNs demonstrated excellent colloidal stability in 5% dextrose, with minimal aggregation. No hemolytic activity was observed at concentrations up to 50 mg/L. Single and repeated administrations revealed no significant changes in body/organ weights, hematological parameters (except transient fibrinogen elevation), or liver/kidney function markers (ALT, AST, BUN, Cr). Histopathology showed preserved tissue architecture in major organs, with mild hepatocyte vacuolation at 30 days. Biodistribution indicated hepatic/splenic accumulation and rapid blood clearance, suggesting hepatobiliary elimination. **Conclusions**: Platinum porphyrin–doped F8BT CPNs exhibited minimal acute and subacute toxicity, favorable biocompatibility, and no systemic adverse effects in murine models. These findings support their potential as safe PS candidates for PDT. However, chronic toxicity studies are warranted to address long-term organ accumulation and metabolic impacts. This preclinical evaluation provides a critical foundation for advancing CPNs toward clinical applications in oncology.

## 1. Introduction

Photodynamic therapy (PDT) has emerged as a viable approach for treating many cancers owing to its minimally invasive characteristics and selective cytotoxicity towards malignant cells [1,2]. This therapy utilizes photosensitizing agents (PSs) that, when activated by specific light wavelengths, produce reactive oxygen species (ROS), resulting in killing of tumor cells [3,4]. Nano-based PSs are advanced materials designed for applications in PDT, cancer treatment, antimicrobial therapy, and surface disinfection, among others. These third-generation PSs leverage the unique properties of nanoparticles (NPs) to enhance the efficiency of photosensitization, such as improved targeting, controlled release, and enhanced light absorption [5]. Notwithstanding its potential, the clinical implementation of PDT encounters obstacles, such as restricted tissue penetration of excitation light [6], inadequate biodistribution of PS [7,8], and possible off-target effects [9,10]. Nano-based PSs may also pose intrinsic toxicity risks that need to be carefully evaluated. The toxicity of nano-based PSs can depend on the materials used, such as metal oxides (e.g., TiO_2_, ZnO), organic polymers, or carbon-based nanomaterials. Some materials may inherently exhibit cytotoxicity or induce inflammatory responses [11,12,13]. NPs may accumulate in organs such as the liver, spleen, or kidneys, leading to potential long-term toxicity [14]. The inability of the body to fully clear these materials can result in chronic inflammation or organ damage [15]. While nano-based PSs hold immense potential for revolutionizing medical and environmental applications, their potential toxicity cannot be overlooked. Rigorous safety assessments are essential to harness the benefits of nano-based PSs while minimizing their adverse effects. 

Conjugated polymer nanoparticles (CPNs) have recently gained attention as next-generation PSs due to their superior optical properties, stability, and tunable surface modifications, which enhance their biocompatibility and tumor-targeting capabilities [16,17,18,19,20,21]. Among the wide range of conjugated polymers (CPs) developed for the synthesis of CPNs, poly(9,9-dioctylfluorene-alt-benzothiadiazole) (F8BT) stands out as an optoelectronically active polymer with promising applications in bioimaging, phototherapy, and drug delivery [22,23,24,25,26]. F8BT is a π-conjugated organic semiconductor polymer composed of alternating 9,9-dioctylfluorene and benzothiadiazole units, exhibiting strong fluorescence, excellent photostability, and efficient charge transport properties, making it an ideal candidate for theranostic applications. The synthesis of NPs from hydrophobic F8BT can be achieved through nanoprecipitation or emulsion-based methods, often requiring amphiphilic stabilizers such as poly(styrene-co-maleic anhydride) (PSMA) or polystyrene grafted with polyethylene glycol (PSPEG), among others, to enhance colloidal stability and biocompatibility [16,27]. These NPs can be further functionalized with targeting ligands, PSs, or therapeutic agents to enhance their selectivity and efficacy in biomedical applications [18,22]. Due to their extraordinary extinction coefficient and high fluorescence quantum yield, F8BT-based CPNs enable real-time tracking in biological systems, offering a versatile platform for imaging-guided therapies, including photodynamic treatment for cancer [28,29,30]. Remarkably, these CPNs can also produce ROS when stimulated by ultrasound in the therapeutic window which allows for deep tissue penetration [31]. Specifically, metallated porphyrin-doped CPNs have demonstrated high photodynamic efficiency against glioblastoma and other aggressive tumors [32,33,34]. In addition, these nano-based PSs have demonstrated efficiency in the photodynamic inactivation of pathogenic microorganisms that are harmful to human and animal health, expanding their range of biomedical applications [31,34,35]. However, comprehensive toxicological characterization is essential to evaluate their biosafety, pharmacokinetics, and potential systemic effects before advancing to clinical applications.

This study presents a preclinical toxicological evaluation of platinum porphyrin–doped F8BT CPNs, assessing their biocompatibility, in vivo distribution, and potential adverse effects under both single-dose and repeated-dose administration protocols. Our findings contribute to establishing the safety profile of these nanomaterials, paving the way for their future development as effective PSs in PDT.

## 2. Materials and Methods

### 2.1. Materials

The fluorescent polymer poly(9,9-dioctylfluorene-alt-benzothiadiazole) (F8BT, Mn = 70,000 g/mol, PDI = 2.4, American Dye Source Inc., Montreal, QC, Canada), the amphiphilic functional polymers poly(styrene-co-maleic anhydride) (PSMA, terminated by cumene, 68% styrene content, average molecular weight approximately 1700 g/mol, Sigma Aldrich, St. Louis, MO, USA), the comb-like polymer polystyrene-polyethylene glycol-functionalized with carboxyl groups (PS-PEG-COOH, backbone Mn = 6500 g/mol, branches Mn = 4600 g/mol, Polymer Source Inc., Dorval, QC, Canada), and the porphyrin Pt(II) octaethylporphyrin (PtOEP, >95%, Frontier Scientific, Newark, DE, USA) were utilized as received. Tetrahydrofuran (THF, pro-analysis grade, Sintorgan, Villa Martelli, Argentina) was employed to dissolve polymers following 5 h reflux with potassium hydroxide pellets (KOH, pro-analysis grade, Sigma-Aldrich, St. Louis, MO, USA). Nanoprecipitation was conducted in double-distilled water, and further purification was performed with an ELGA PURELAB Classic UV system (∼18.2 MΩ/cm) to eliminate ions, organic substances, and particulate matter using a 0.2 μm pore diameter filter.

### 2.2. Nanoparticle Synthesis

CPNs were developed using a previously reported nanoprecipitation protocol [33,36]. Briefly, a stock solution of F8BT was prepared by dissolving the polymer in THF to a concentration of ~500 mg/L. This solution was filtered with a 0.2 μm pore size PTFE membrane syringe filter (Iso-Disc, Sigma-Aldrich) to remove undissolved polymers. The concentration of the filtered solution was recalculated from its absorption spectrum using the known absorption coefficient (45.4 g^−1^ L cm^−1^ in THF at 456 nm). Stock solutions of PSMA (2 g/L), PS-PEG-COOH (2 g/L), and PtOEP (0.25 g/L) in THF were also prepared. Afterwards, F8BT, PtOEP and PSMA, or PS-PEG-COOH were mixed in THF to final concentrations of 50, 5, and 10 mg/L, respectively. A volume of 5 mL of F8BT/PSMA or PS-PEG-COOH/PtOEP solution was quickly injected into 10 mL of milliQ water while sonicating (PS-30A, Arcano, Buenos Aires, Argentina), and the resulting mixture was further sonicated for 10 min. THF was removed under reduced pressure in a rotary evaporator, yielding a final volume of 10 mL. The injection procedure was repeated two more times with two equal mixed polymer solutions in THF (5 mL; F8BT, PSMA, or PS-PEG-COOH and PtOEP) with complete THF removal after every THF mixed solution injection. This process yielded a concentrated CPN suspension. Finally, the obtained NP dispersion was filtered through a 0.2 μm pore size cellulose acetate membrane filter (25 mm, gamma sterile, Micron Separations Inc., Westborough, MA, USA) to eliminate large aggregates. CPN concentration after filtering is expressed in terms of F8BT mass concentration (mg/L) and was calculated using the absorption coefficient of neat F8BT CPN (37.1 g^−1^ L cm^−1^ in water at 456 nm). Different stock batches of CPN suspensions were prepared with final a F8BT concentration of ~150 mg/L. CPNs were characterized by dynamic light scattering (DLS, Malvern 4700), absorption (Agilent Hewlett Packard, HP 8452A, Agilent Technologies, Inc., Santa Clara, CA, USA), and emission spectroscopy (Horiba, Fluoromax-4, Horiba Scientific, Edison, NJ, USA). CPNs synthesized using F8BT, PtOEP, and PSMA were designated as CPN-PSMA-PtOEP, while those prepared with F8BT, PtOEP, and PS-PEG-COOH were referred to as CPN-PSPEG-PtOEP.

### 2.3. Dynamic Light Scattering (DLS) Size Characterization of CPNs

Measurements were performed using a Malvern 4700 instrument at 25 °C. Light scattering results were analyzed using Zetasizer software (Zetasizer 6.x, provided by the instrument manufacturer) to obtain hydrodynamic radius number distributions. CPN suspensions for analysis were prepared with MilliQ water filtered through 0.2 μm pore size filters prior to data acquisition. Measurements were performed in a 1 cm quartz cuvette.

### 2.4. Assessment of the Colloidal Stability of CPNs in Various Parenteral Administration Solutions by DLS

CPNs were prepared using PS-PEG-COOH and PSMA stabilizer polymers, both doped with PtOEP, at a final concentration of 150 mg/L. Furthermore, three different isotonic solutions for medical applications were developed to suspend the CPNs: PBS 1x, NaCl 0.9%, and dextrose 5%. These isotonic solutions are routinely employed for parenteral drug administration in in vivo studies. The CPN formulations dispersed in the different media were placed in quartz cuvettes to assess particle hydrodynamic diameter via DLS. Measurements were conducted at intervals of 0 min, 30 min, and 1 h. This approach was aimed to evaluate colloidal stability across different isotonic media for subsequent CPN administration trials in animals.

### 2.5. Animals Care

Healthy adult (~6 weeks old) BALB/c mice (*Mus musculus*) weighing between 25 and 35 g, with sex-dependent variations, were obtained from the animal facility of the Faculty of Exact, Physical-Chemical, and Natural Sciences at the National University of Río Cuarto (UNRC). The mice were housed in plastic cages with bedding in a ventilated, temperature-controlled, and standardized sterile animal room. They were maintained under a 12/12 h light/dark cycle, with food and water available ad libitum. All animal procedures were strictly in compliance with the Guide for the Care and Use of Laboratory Animals published by the NIH and approved by the Research Ethics Committee (COEDI) of UNRC, Río Cuarto, Argentina (approval registration code Nº 300/21).

All administrations and subsequent toxicity assessments were conducted under standard laboratory lighting (ambient light, no specific wavelength restriction). While this setup does not involve deliberate photoactivation (e.g., controlled irradiation at specific wavelengths), we acknowledge that ambient light exposure could theoretically introduce unintended photodynamic effects.

### 2.6. Hemolysis Assay

Blood was collected from BALB/c mice (6 weeks old, *n* = 3) under inhalational anesthesia with 2% isoflurane, followed by euthanasia via decapitation. The blood was stored in potassium EDTA collection tubes and repeatedly washed with phosphate-buffered saline (PBS) to remove free hemoglobin. Red blood cells (RBC) were exposed to different concentrations of CPN-PSMA-PtOEP (1, 2, 5, 10, 25, and 50 mg/L) and incubated at 37 °C for 1 h. The following controls were established according to Nanotechnology Characterization Laboratory (NCL) recommendations [37]. Positive Control: Triton X-100 at a stock concentration of 1% (10 mg/mL) was used to induce hemolysis, as it causes lysis of the cytoplasmic membrane of RBCs. Negative Control: RBCs were exposed to sterile PBS at room temperature. Vehicle Control: This served as a control for the vehicle or medium used to formulate the NPCs, which in this case was 5% dextrose. No-Blood Control: CPNs diluted in PBS at the same final concentration as those tested in the blood assay were used as a control to rule out false-positive results. After incubation, the samples were centrifuged, and the absorbance was measured at 540 nm using a Multiskan FC microplate reader (Thermo Scientific, Waltham, MA, USA).

### 2.7. Animal Treatments and Sample Collection

#### 2.7.1. Single-Dose Toxicity

**Figure 1:**
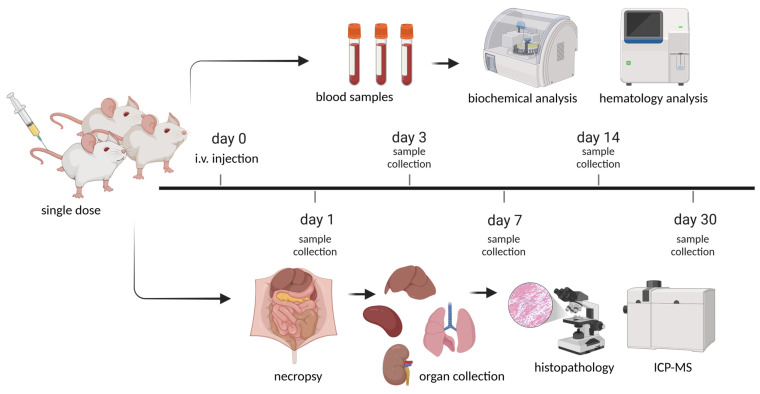
Schematic representation of the experimental timeline for the single-dose toxicity study of CPNs in BALB/c mice. A single intravenous (i.v.) injection was administered at day 0, followed by sample collection at different time points. Blood and organ samples were collected on days 1, 3, 7, 14, and 30 post-euthanasia for histopathological and biochemical analyses. The study aimed to assess the systemic and organ-specific toxicity of the CNPs over time. Created in BioRender (https://www.biorender.com/, accessed on 1 March 2025). Ibarra, L. (2025) https://BioRender.com/f26a222.

For single-dose toxicology study of CPNs, doses of 0.3 and 1 mg (CPN)/kg (mouse) were chosen based on previous pilot study of the efficacy of CPN-PDT and the regulatory guide of the Nanotechnology Characterization Laboratory (NCL) [33,37]. Intravenous (i.v.) injections of CPN suspended in dextrose 5% were conducted through the mouse tail vein. Intravenous injections of sterile dextrose 5% were also given to mice as controls. The number of animals used in this evaluation followed OECD (2001) guidance and reduction principles [XX], i.e., using the lesser possible number of animals to obtain statistical relevance [38]. Mice were randomly divided into different groups based on the administration of CPN-PSPEG-PtOEP and CPN-PSMA-PtOEP at doses of 0.3 and 1 mg/kg or dextrose 5% for the control group. After administering the CPN samples, the animals were closely monitored for the first hour, followed by periodic observations over the next 4 h. Subsequently, they were examined every 12 h for the following days. Throughout the study, clinical assessments were conducted to detect mortality, behavioral or neurological changes, and other abnormalities, while weight was recorded weekly until the end of the experiment. Each group had six mice (three male and three female), and different biochemical parameters were determined after blood collection (potassium EDTA collection tube) at 1, 3, 7, 14, and 30 days after a single i.v. dose of CPNs (Figure 1).

#### 2.7.2. Repeated-Dose Toxicity

**Figure 2:**
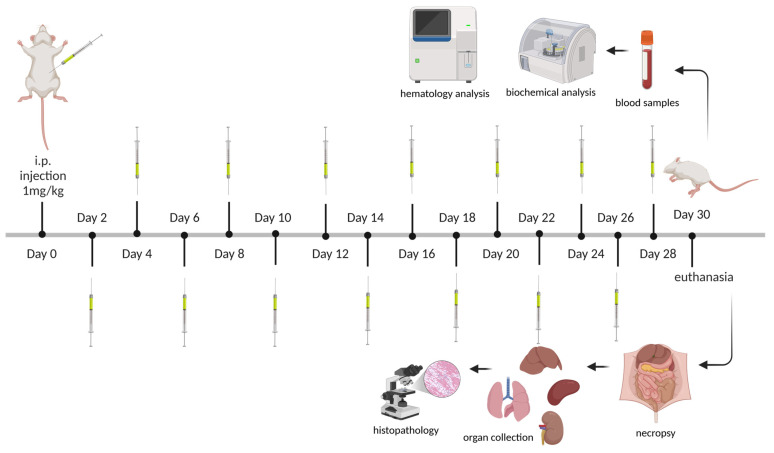
Schematic representation of the experimental timeline for the subacute toxicity study of CPNs. BALB/c mice received intraperitoneal (i.p.) injections of CNPs at a dose of 1 mg/kg every 48 h over a 28-day period. Blood and organ samples were collected at the end of the study (day 30) and at selected time points for hematological and biochemical analyses. Throughout the study, clinical and physiological assessments were performed. Created in BioRender (https://www.biorender.com/, accessed on 1 March 2025). Ibarra, L. (2025) https://BioRender.com/f26a222.

A particular drug dose can effectively reduce disease symptoms with minimal toxicity over a short period of time. However, adverse effects resulting from prolonged exposure remain a key factor in the withdrawal of pharmaceuticals from the market [39,40]. To evaluate the repeated-dose toxicity of CPNs, twenty-four mice (12 males and 12 females), aged ~6 weeks with a body weight of 30 ± 5 g, were randomly assigned to groups. Mice in the treatment groups received intraperitoneal (i.p.) doses of 0.1, 0.5, or 1 mg/kg every 48 h for 28 days, while the control group received 5% dextrose as a vehicle (Figure 2). Antisepsis of the abdominal area was performed using 70% alcohol prior to the i.p. injections. Following the injections, body weight and clinical manifestations were documented at certain time points in accordance with regulations. Symptoms and deaths were meticulously documented during the entire study. Two days after the final dose administration, the mice were euthanized under inhalational anesthesia. Blood and organ samples were collected for toxicological analysis.

### 2.8. Serum Collection, Hematology Analysis, and Biochemical Determination

Prior to euthanasia, the mice were anesthetized with 2% isoflurane (inhalational anesthesia). Once unconsciousness was achieved, the animals were euthanized by decapitation, and the total blood volume was collected in 1.5 mL tubes. Centrifugation at 1200 rpm for 5 min was performed to separate the formed elements (red blood cells, white blood cells, and platelets). The serum was collected and stored at −80 °C until further analysis. Liver function was evaluated via serum levels of alanine aminotransferase (ALT) and aspartate aminotransferase (AST). Nephrotoxicity was determined by blood urea nitrogen (BUN) and creatinine (Cr) serum levels. These parameters were all assayed using a biochemical autoanalyzer (Cobas c 311, Roche Diagnostics, Rotkreuz, Switzerland). The evaluated hematological parameters included the erythrocyte series (red blood cell count, hemoglobin concentration (Hb), hematocrit (HTC), mean corpuscular volume (MCV), mean corpuscular hemoglobin (MCH), and mean corpuscular hemoglobin concentration (MCHC)), the leukocyte series (total leukocyte count, segmented neutrophils, lymphocytes, monocytes, and eosinophils), and additional parameters such as plasma proteins, fibrinogen, and platelet count. These parameters were determined using an Abacus Junior 30 hematology analyzer (STRATEC SE, Birkenfeld, Germany).

### 2.9. Histopathological Examinations

Tissues samples from spleen, liver, kidney and lung recovered from the necropsy from single-dose and repeated dose toxicity studies were fixed in 4% formalin, embedded in paraffin, sectioned at 5–10 μm, and stained with hematoxylin and eosin (H&E) for histological examination using standard techniques. After hematoxylin eosin staining, the slides were observed, and photos were taken using Zeiss Primo Star LED Binocular Microscope (ZEISS, Oberkochen, Baden-Württemberg, Germany). The pathologist was blinded to the identity and analysis of the pathology slides.

### 2.10. Biodistribution and Blood Clearance Analysis of CPNs Using ICP-MS

To evaluate the biodistribution and blood clearance of CPN-PSPEG-PtOEP and CPN-PSMA-PtOEP administered i.v. to mice, the accumulated CPNs in various organs and their elimination kinetics from the bloodstream were determined at different times post-administration. Mice received a dose of 1 mg/kg of CPN-PSPEG-PtOEP or CPN-PSMA-PtOEP and were euthanized 1, 3, or 7 days post-administration. Following euthanasia, a necropsy was performed, and organs such as the liver, spleen, kidney, and lungs were collected for analysis. Tissue samples were first mechanically homogenized and then digested under acidic conditions using a microwave reactor. Quantification of CPNs in the tissues was conducted using Inductively Coupled Plasma Mass Spectrometry (ICP-MS, PerkinElmer, NexION 300X, Shelton, CT, USA), a highly sensitive technique for detecting trace heavy metals, ionizing samples with high-temperature argon plasma, and analyzing them via mass spectrometry. The analysis focused on the detection of platinum (Pt, μg/kg (sample)), a component of PtOEP, to estimate the accumulation of CPNs in the different organs. Three of the most abundant Pt isotopes were evaluated (Pt-194, Pt-195, Pt-196). To further analyze CPN blood clearance, mice (*n* = 3) were administered CPN-PSMA-PtOEP i.v. at a dose of 1 mg/kg, and blood collection was performed 0, 3, 6, 12, and 24 h post-administration. Quantification of fortified blanks with a Pt standard and fortified samples with Pt after digestion was performed to evaluate matrix effects. The recovery of Pt was consistently above 91%. The detection limit for Pt in tissue samples was 1 ppb (μg/kg).

### 2.11. Statistical Analysis

Data are presented as mean values ± standard deviation. Statistical analyses were conducted utilizing GraphPad Prism software version 8.0 (GraphPad Software, San Diego, CA, USA). Kolmogorov–Smirnov normality tests were utilized to compare unpaired groups further. The experimental design involved comparing means between distinct groups of numerical variables using one-way or two-way analysis of variance (ANOVA) and Tukey’s test. The criterion for statistical significance was established at *p* < 0.05.

## 3. Results

### 3.1. Colloidal Stability Evaluation of CPNs in Parenteral Administration Solutions Using DLS

To evaluate the suitability of CPNs for parenteral administration, their colloidal stability was assessed in various clinically relevant isotonic infusion solutions using DLS. The stability of CPN suspensions is a critical parameter influencing their biodistribution, efficacy, and safety in vivo. Therefore, CPN-PSPEG-PtOEP and CPN-PSMA-PtOEP samples were incubated in different parenteral media, and changes in hydrodynamic diameter (d_h_) and polydispersity index (PDI) were monitored over time. Both stabilizer polymers were added in equal mass proportions relative to the mass of F8BT, ~20% *w*/*w*. We observed that CPN-PSMA-PtOEP had an average d_h_ of 18 ± 3 nm in pure water, with a PDI of 0.24, whereas CPN-PSPEG-PtOEP exhibited a larger d_h_ of 29 ± 4 and a PDI of 0.25 (Figure S1). These findings are consistent with previous results [18].

CPN suspensions in isotonic infusion solutions (0.9% NaCl, 5% dextrose, and PBS 1X) were assessed at distinct intervals (pre-preparation, immediately post-preparation, at 30 min, and at 1 h) utilizing the DLS technique to ascertain alterations in size distribution and potential aggregation and/or precipitation resulting from increased ionic strength, which would be indicated by a larger d_h_. The results for CPN-PSPEG-PtOEP and CPN-PSMA-PtOEP are presented in Table 1 and Table 2, respectively.

CPNs suspended in 5% dextrose solutions exhibited minimal variation at a concentration of 150 mg/L for both stabilizers used in the synthesis. We observed that CPN-PSMA-PtOEP exhibited superior colloidal stability over time, as indicated by only slight modifications in d_h_ compared to CPN-PSPEG-PtOEP. The behavior of the different CPNs in the isotonic solutions allows us to identify 5% dextrose solution as a suitable vehicle for CPN administration injections in further studies.

### 3.2. Hemolytic Activity of CPNs

**Figure 3:**
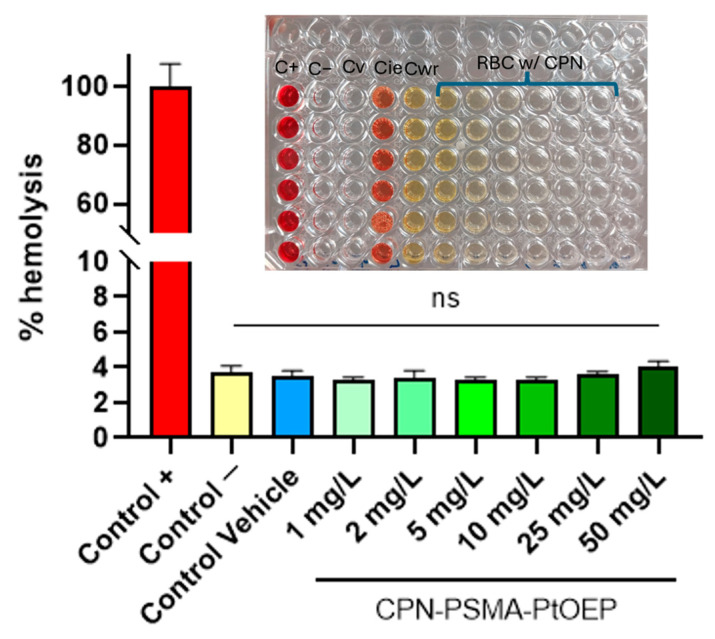
Percentage of hemolysis induced by CPN-PSMA-PtOEP at different concentrations. C+: positive control with Triton X100; C−: negative control with PBS; Cv: vehicle control of CPNs (5% dextrose); Cie: inhibition/enhancement control; Cwr: blood-free control. ns = no statistically significant differences.

The aim of this test was to assess the hemolytic capacity of CPN-PSMA-PtOEP employing BALB/c mouse blood. Hemolysis serves as a vital indication of NP biocompatibility, since the lysis of red blood cells can lead to significant detrimental consequences in the body [37]. The findings indicated that CPN-PSMA-PtOEP did not cause hemolysis at any of the evaluated concentrations (1, 2, 5, 10, 25, and 50 mg/L) (Figure 3). The absorbance values at 540 nm of samples treated with CPNs was similar to that of the negative control (PBS) and the vehicle control (5% dextrose), suggesting no substantial hemoglobin release. Conversely, the positive control (Triton X-100 1%) exhibited elevated absorbance values, thus validating the assay’s efficacy in identifying hemolysis.

### 3.3. Single-Dose Toxicity of CPNs

For the single-dose toxicity assay, five groups of adult BALB/c mice (*n* = 25 per group) were administered a single i.v. dose of either 0.3 mg/kg or 1 mg/kg of CPN-PSPEG PtOEP or CPN-PSMA PtOEP. An additional control group received only the vehicle solution of dextrose 5%. The effects of CPN administration were evaluated at 1, 3, 7, 14, and 30 days. Body weight, clinical behavior, blood biochemistry, and histopathology were analyzed. Body weight and organ weights were assessed to evaluate potential toxicological effects of the treatments. The initial body weights were approximately 30 g for males and 25 g for females, with no significant differences in growth curves across treatment groups throughout the study (*p* = 0.9339) (Figure S2). In addition, no significant statistical differences were observed in other organ weights, including the liver, spleen, lung, and kidneys among different CPN-treated groups and the control group (Figure S2). These results suggest that CPN-PSPEG PtOEP and CPN-PSMA PtOEP do not significantly impact the growth and development of mice. Furthermore, clinical examination revealed no signs of toxicity, such as fatigue, anorexia, lethargy, or neurological alterations.

#### 3.3.1. Blood Hematology Analysis

**Figure 4:**
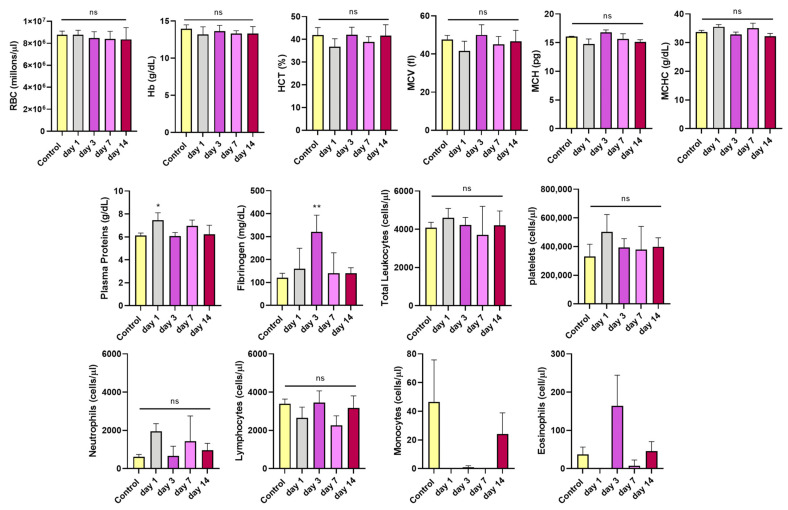
Blood hematological parameters of mice treated with CPN-PSMA-PtOEP (1 mg/kg) and control mice, as determined using an autoanalyzer. Each group has six mice. The data are presented as mean ± SD. Significant changes were determined compared with the control group (* *p* < 0.05; ** *p* < 0.01; ns = no statistically significant differences).

To assess the effects of i.v. administration of CPN-PSMA-PtOEP, hematological analyses were performed at 1, 3, 7, and 14 days after single-dose of CPNs. Results indicated that all measured parameters in mice treated with CPN-PSMA-PtOEP (1 mg/kg) remained within normal ranges for the specie and were comparable to those of the control group (Figure 4). The stability of RBC levels, HTC, Hb, MCV, MCH, and MCHC suggests that these NPs are safe and biocompatible, at least within the context of the evaluated hematological parameters. Furthermore, to assess the safety of CPNs, not only hematological parameters but also immune system–related markers were considered, including total leukocytes, neutrophils, lymphocytes, monocytes, eosinophils, and platelets. The analysis demonstrated that the administration of CPN-PSMA-PtOEP (1 mg/kg) did not negatively affect immune-related hematological parameters in healthy BALB/c mice.

In addition, plasma protein levels were analyzed, with a particular focus on fibrinogen. While total protein levels remained within normal ranges across all time points, a slight increase was observed on day 1 after CPN administration compared to the control group. Fibrinogen levels exhibited a transient increase. Specifically, at day 3, fibrinogen levels were significantly higher in the treated group compared to controls (*p* = 0.0062), suggesting a potential initial inflammatory response or reactive coagulation process following CPN administration. However, by day 14, fibrinogen levels returned to normal, indicating that this response was temporary, and that homeostasis was restored without long-term complications.

#### 3.3.2. Serum Biochemistry After Single-Dose I.V. Administration

**Figure 5:**
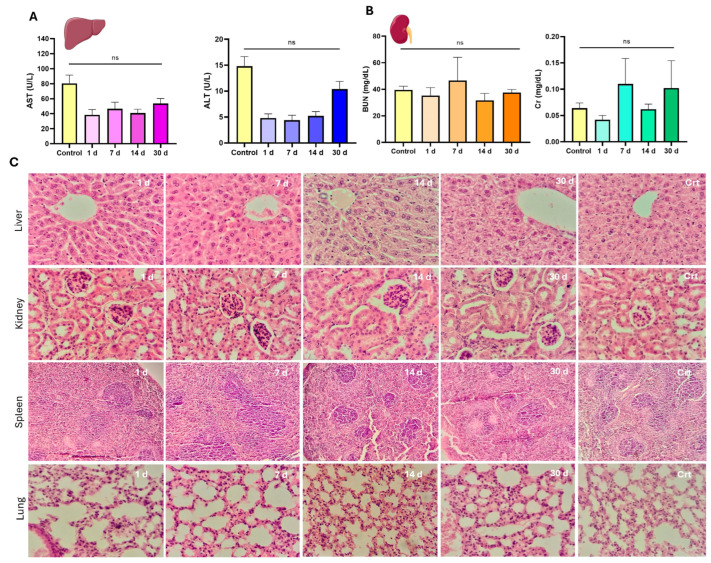
Serum biochemistry and histopathological evaluation of major organs following a single dose of CPNs. Serum biochemical markers associated with kidney function (**A**), including blood urea nitrogen (BUN) and creatinine (Cr), as well as liver function markers (**B**), including aspartate aminotransferase (AST) and alanine aminotransferase (ALT). (**C**) Representative hematoxylin and eosin (H&E)-stained sections of liver, kidney, spleen, and lung tissues collected at different time points (1, 7, 14, and 30 days) post-administration of CPNs. Control (Crt) samples are included for comparison (ns = no statistically significant differences). Magnification 400×.

Serum biochemistry analyses were conducted to evaluate the effects of CPN-PSPEG-PtOEP and CPN-PSMA-PtOEP at different doses (0.3 and 1 mg/kg) on the biological functions of major detoxifying organs, such as the liver and kidneys. The results for CPN-PSMA-PtOEP are presented in Figure 5. In the functional renal assessment, BUN and Cr serum levels following administration of CPNs remained within the reference range for the species, with slight decreases observed at days 1, 14, and 30 for Cr serum levels (Figure 5B). However, statistical analysis showed no significant differences compared to the control group (*p* = 0.1294). Furthermore, serum biochemistry analysis of ALT and AST levels indicated normal liver function across all experimental groups. No significant deviations were observed in ALT or AST levels following the administration of CPN-PSMA-PtOEP at either dose (0.3 and 1 mg/kg) compared to the control group (Figure 5A). Treatment with CPN-PSPEG-PtOEP followed the same tendency (Figure S3). These findings suggest that the tested CPNs did not induce hepatic toxicity or impair liver function within the evaluated timeframe.

#### 3.3.3. Histopathological Assessment of Major Organs

Histopathological evaluation of liver, kidney, spleen, and lung tissues revealed no significant pathological alterations across the CPN-PSMA-PtOEP group compared to control group. Organs from the control group exhibited normal histological architecture, with no evidence of degenerative damage, inflammatory responses, or circulatory disturbances. Similarly, liver tissue from treated groups maintained structural integrity, with hepatocytes displaying a typical hexagonal lobular arrangement and Kupffer cells appearing without abnormalities. No signs of biliary cholestasis, fibrosis, or inflammatory infiltrates were observed at any time point. However, in the group evaluated 30 days post-injection, mild hydropic degeneration was detected in hepatocytes, characterized by the presence of small, pale vacuoles indicative of increased water uptake in the endoplasmic reticulum (Figure 5C). This finding, while not indicative of overt toxicity, may be attributed to CPN accumulation. Microscopic evaluation of renal tissue morphology across all experimental groups revealed a well-preserved parenchymal structure with an appropriate cortex-to-medulla ratio. In the renal cortex, glomeruli appeared well-developed, and both proximal and distal convoluted tubules exhibited well-formed epithelial cells consistent with normal parenchymal architecture, without any signs of degenerative or proliferative disorders (Figure 5C). In the stromal tissue of both the cortex and medulla, no inflammatory cell infiltration was observed, ruling out the presence of interstitial or suppurative glomerulonephritis. Additionally, blood vessels displayed clear lumens with no evidence of circulatory disturbances. Similarly, splenic tissue exhibited the same characteristics as the control group. The organs displayed well-developed capsules with connective tissue trabeculae extending into the parenchyma. The lymphoid follicles were well formed, composed of mature and immature lymphoid cells, with no signs of immune system alteration such as lymphoid depletion or hyperplasia (Figure 5C). Finally, lung tissues from the treated groups showed no pathological alterations. Alveolar septa were thin and composed of pneumocytes and capillary networks, with no evidence of inflammatory cell infiltration. Additionally, the alveolar spaces remained clear, with no signs of exudative inflammatory cells (polymorphonuclears leukocytes), further confirming the absence of pulmonary pathology (Figure 5C). Overall, these results suggest that the administered CPNs do not induce significant histopathological changes in major detoxifying organs within the studied timeframe.

### 3.4. Biodistribution Analysis and Plasma Kinetics of CPNs by ICP-MS

**Figure 6:**
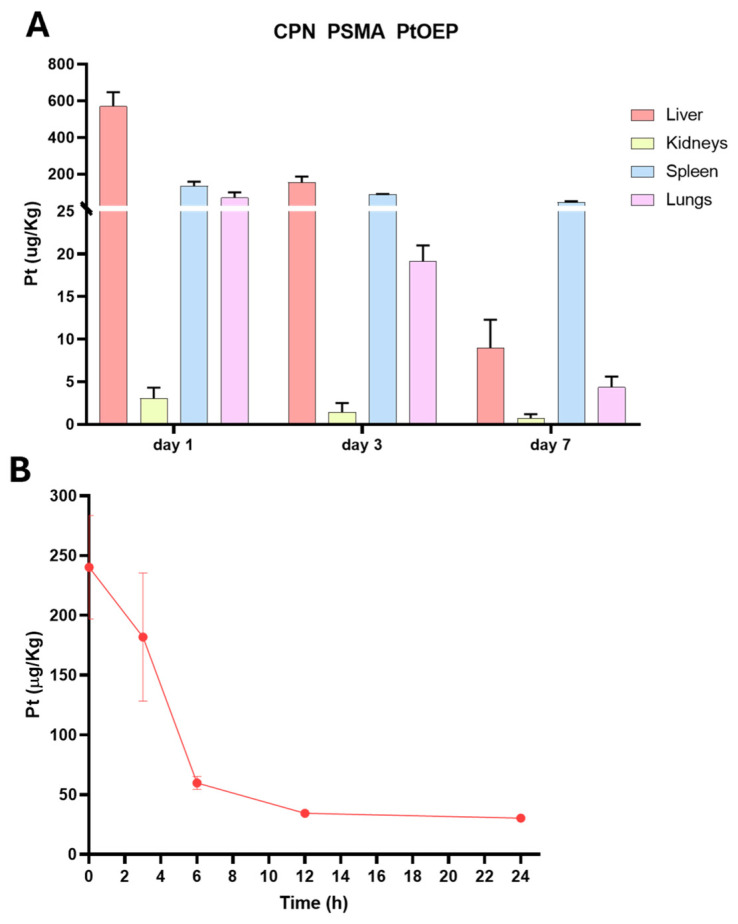
Biodistribution and blood content of CPN PSMA PtOEP. (**A**) Biodistribution of Pt-containing CPNs in major organs (liver, spleen, lungs, and kidneys) at 24 h, 3 days, and 7 days post-administration of 1 mg/kg i.v. The liver, spleen, and lungs exhibited the highest Pt accumulation at 24 h, followed by a decline at day 3 and day 7. Kidney accumulation remained minimal, indicating that renal clearance is not the primary elimination route. (**B**) Blood circulation profile of CPN PSMA PtOEP over time, showing a progressive decrease in Pt concentration, suggesting systemic clearance following administration. Data are presented as mean ± standard deviation.

ICP-MS was employed to quantitatively determine the Pt content provided by PtOEP in CPNs across tissue samples, including liver, kidney, spleen, and lung. This analysis was performed on BALB/c mice (*n* = 3) that received a single i.v. dose of 1 mg/kg of either CPN-PSMA-PtOEP or CPN-PSPEG-PtOEP. Tissue samples were collected at 1, 3, and 7days post-administration. Following the administration of CPN-PSMA-PtOEP, Pt levels were highest in the liver, spleen, and lungs at 24 h, suggesting predominant uptake by the mononuclear phagocyte system (Figure 6A). A subsequent decline in Pt levels was observed at day 3, followed by a decrease at 3 and 7 days, which may indicate nanoparticle redistribution or prolonged retention within these organs. In contrast, kidney tissue exhibited minimal Pt accumulation throughout the study, suggesting that the primary route of elimination is not renal but rather hepatobiliary. This biodistribution pattern highlights the potential influence of CPN uptake and hepatic processing on nanoparticle clearance dynamics.

The concentration of Pt-containing CPN in the bloodstream progressively decreased over time (Figure 5B). The highest levels were detected at the earliest time point, with a rapid decline by 6 h, indicating effective clearance from circulation. After 12 h, only minimal Pt levels remained in the blood, supporting the hypothesis of tissue redistribution and elimination predominantly through non-renal pathways.

### 3.5. Repeated-Dose Toxicity of CPNs

**Figure 7:**
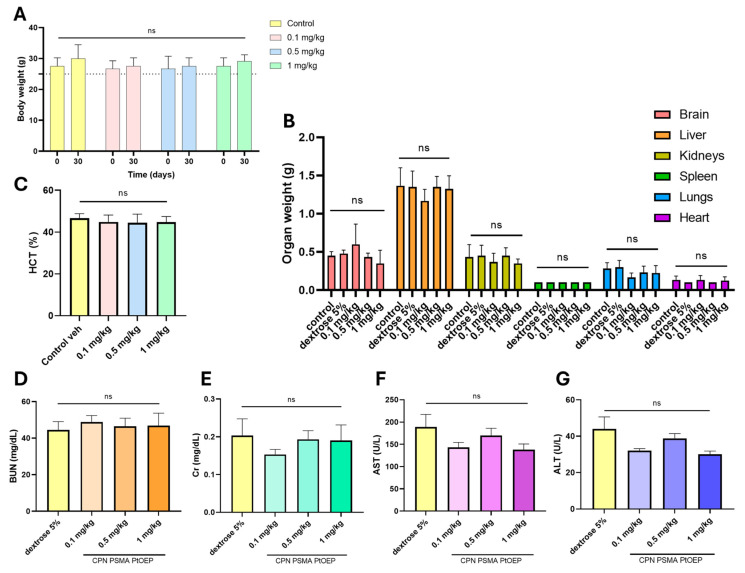
Evaluation of repeated-dose toxicity following intraperitoneal administration of CPNs. Mice were treated with 0.1, 0.5, or 1 mg/kg of CPN-PSMA-PtOEP every 48 h for 28 days, while the control group received 5% dextrose. (**A**) Body weight progression of mice across different treatment groups. (**B**) Relative organ weights of key metabolic and excretory organs, including the liver, spleen, and kidneys. (**C**) Hematocrit percentage across treatment groups. Serum biochemical markers associated with kidney function, including blood urea nitrogen (BUN) (**D**) and creatinine (Cr) (**E**), as well as liver function markers, including aspartate aminotransferase (AST) (**F**) and alanine aminotransferase (ALT) (**G**). No statistically significant differences (ns) were observed among the groups, indicating that CPN administration did not induce overt toxicity. Data are presented as mean ± standard deviation. *p*-values were determined using one-way ANOVA.

Clinical observations revealed no overt signs of distress, adverse reactions, or mortality across all groups, suggesting that repeated CPN exposure did not produce immediate toxicity. Moreover, no statistically significant variations were found in the weights of animals between the beginning day and the final time point (30 days) across all groups, including various dose groups and the control group (*p* = 0.9842) (Figure 7A). No weight loss or decreased weight gain indicative of discomfort was noted. Upon conclusion of the study, animals were euthanized to obtain various samples (blood and organ tissues) for analysis. Initially, the organs were weighed to compare them with the control group and evaluate any changes. Each organ was evaluated individually across the various dose groups and the control group to identify any discrepancies; however, no statistically significant changes were noted in any instance (*p* = 0.8314) (Figure 7B).

At the end of the study (30 days), blood samples were collected for analysis. HTC levels remained within physiological ranges across all treatment groups, with no statistically significant differences observed (Figure 7C). Serum biochemical markers associated with kidney and liver function were assessed to evaluate potential systemic toxicity. BUN (Figure 7D) and Cr (Figure 7E) levels were measured as indicators of renal function, while AST (Figure 7F) and ALT (Figure 7G) were analyzed as markers of hepatic function. No significant differences in BUN or Cr levels were detected between treatment groups and the control, suggesting that repeated administration of CPNs did not induce renal dysfunction. Similarly, AST and ALT levels remained within physiological ranges across all groups, with no notable deviations from the control group. These findings indicate that prolonged exposure to CPNs at the tested doses does not result in detectable hepatotoxicity or nephrotoxicity under the study conditions.

**Figure 8:**
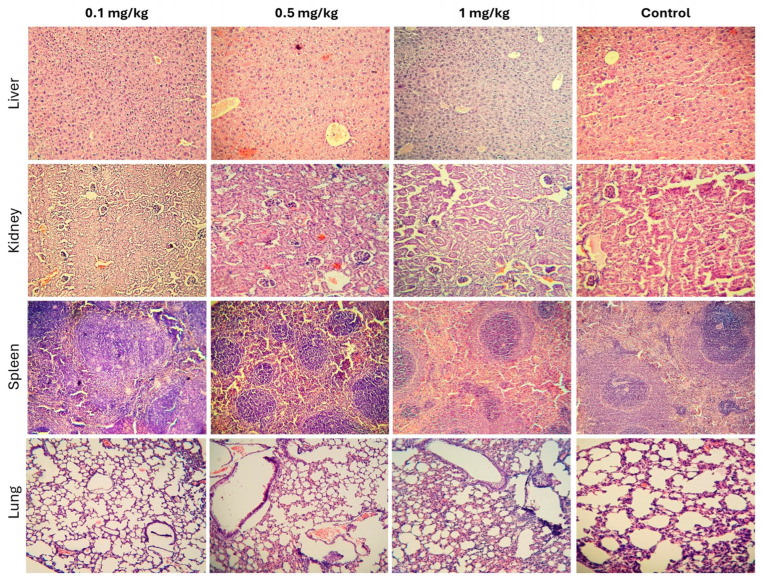
Histopathological analysis of major organs following repeated administration of CPNs. Representative hematoxylin and eosin (H&E)-stained sections of liver (**top row**), kidney (**second row**), spleen (**third row**), and lung (**bottom row**) tissues from control and treated groups (0.1, 0.5, and 1 mg/kg). No signs of inflammation or tissue damage attributable to CPN administration were observed across all groups. Magnification 250×.

Histopathological analysis of liver tissues from all experimental groups (control, 0.1, 0.5, and 1 mg/kg) revealed well-preserved hepatic architecture, with hepatocytes arranged in characteristic Remak trabeculae. The hepatocytes exhibited well-developed nuclei with appropriately distributed chromatin, and the cytoplasm showed only mild hydropic degeneration across all groups, with no significant differences. No signs of inflammatory cell infiltration or structural alterations indicative of hepatic toxicity were observed (Figure 8, top row). Renal tissue evaluation demonstrated a well-maintained corticomedullary ratio and normal parenchymal organization across all groups. Tubular epithelial cells in both the cortex and medulla exhibited normal nuclear and cytoplasmic morphology, without signs of damage. A slight increase in Bowman’s capsule filtration space was observed in the 1 mg/kg group; however, this change was not significant and was consistent with serum biochemical markers of kidney function (BUN and Cr). No evidence of inflammatory cell infiltration or exudation suggesting renal inflammation was detected (Figure 8, second row). The spleen exhibited normal parenchymal development across all groups, with no pathological alterations. A clear distinction between the white pulp (lymphoid cells) and red pulp (vascular structures) was observed. Reactive germinal centers, characterized by increased size and proliferating lymphoid cells, were present in both the highest-dose and control groups, suggesting normal immune activity (Figure 8, third row). Lung tissue analysis showed occasional alveolar septal ruptures in most samples, including controls, likely due to the euthanasia method used. Additionally, some in-stances of venous congestion, characterized by increased erythrocytes in venous blood vessels, were noted (Figure 8, bottom row). Overall, the histopathological findings suggest that repeated administration of CPNs at the tested doses does not induce significant structural or inflammatory alterations in the examined tissues.

## 4. Discussion

Semiconducting polymers like F8BT belong to a class of photoactive polymers characterized by highly π-conjugated backbones. Their extended π-conjugation combined with intrinsic conformational disorder results in the formation of varying size chromophoric units during CPN synthesis which provide a broad tunability range for the HOMO-LUMO bandgap. This energy difference, referred to as the bandgap, governs fundamental properties such as optical absorption, electrical conductivity, and the photoinduced physical and chemical reactivity of the material. Platinum porphyrin units, when embedded in the CP matrix, can serve as efficient energy traps. When the CP absorbs light, the energy can be transferred to the platinum porphyrin units and then efficiently transfer the energy to molecular oxygen, converting it into ROS such as singlet oxygen. This process is often more efficient than direct energy transfer from the CP alone [19,36]. However, a drawback of these materials lies in their highly π-conjugated and hydrophobic backbones, which can result in poor aqueous stability and biological compatibility, potentially hindering their clinical utility [41]. While numerous studies highlight CPNs as promising diagnostic probes and therapeutic agents, very few investigations have systematically addressed the toxicological profiles of CP-derived NPs, particularly in biologically relevant systems [42]. Nevertheless, advancing these materials toward biomedical applications requires rigorous, standardized assessments of cytotoxicity, long-term biosafety, and immune interactions—areas that remain underexplored. Our study contributes to addressing this gap by specifically evaluating the toxicity of the synthesized CPNs, underscoring the need for further systematic investigations to establish their in vivo safety for this and other types of nanomaterials with potential medical application.

The preclinical toxicological evaluation of platinum porphyrin–doped F8BT CPNs presented in this study provides critical insights into their biocompatibility and safety profile, addressing key concerns for their potential application in PDT. The findings demonstrate that CPNs exhibit minimal acute and subacute toxicity in murine models, supporting their candidacy for further clinical development. The colloidal stability of CPNs in clinically relevant isotonic solutions, particularly 5% dextrose, underscores their suitability for parenteral administration. The excellent stability of CPN-PSMA-PtOEP over time, with negligible aggregation, aligns with previous reports on PEGylated or amphiphilic polymer–stabilized nanoparticles, which resist opsonization and prolong circulation in vivo [43]. This stability is critical for ensuring predictable biodistribution and minimizing aggregation-related toxicity in vivo. Consequently, the selection of an appropriate stabilizer polymer is a pivotal consideration when designing CPNs for biomedical applications. For instance, Yuan et al. investigated the stability of PFVBT stabilized with N_3_-PEG-NH_2_, demonstrating that PFVBT@N_3_-PEG-NH_2_ retained consistent fluorescence intensity and hydrodynamic diameter after 7 days of incubation in water, PBS, and DMEM cell medium [44]. These findings underscore the necessity of systematically evaluating colloidal stability across diverse isotonic solutions for each CP and stabilizer combination, as variations in polymer chemistry and stabilizer properties may profoundly influence CPN behavior under physiological conditions.

Importantly, the absence of hemolytic activity at CPNs concentrations up to 50 mg/L suggests excellent blood compatibility, a critical advantage over some metal-based or inorganic nanoparticles (e.g., silver or zinc oxide NPs) that exhibit dose-dependent hemolysis [45,46]. This property reduces the risk of anemia or thrombotic complications, enhancing their translational potential.

The current body of published literature on the biocompatibility of CPNs remains limited, with a notable scarcity of foundational research exploring their interactions across biological hierarchies—spanning organismal, tissue, cellular, and biofluid levels. Prior investigations have established that engineered nanomaterials frequently induce adverse biological outcomes, including deleterious effects on discrete blood constituents and disruption of critical physiological processes such as coagulation. For example, single- and multi-walled carbon nanotubes, aminated or carboxylated polystyrene beads, and silica nanoparticles have been shown to exhibit hemolytic activity, promote platelet activation, and trigger aggregation, underscoring their potential to interfere with hemostatic regulation [47,48]. Khanbeigi et al. conducted a systematic evaluation of stealth-configured poly(phenylene vinylene) (PPV) and poly(phenylene ethynylene) (PPE) CPNs, specifically examining their interactions with human blood components. In vitro incubation of CPNs with whole blood and isolated platelets revealed no detectable platelet activation, elevation in platelet–monocyte aggregate formation, or induction of aggregatory responses. Notably, at concentrations exceeding 150 mg/L, CPNs induced approximately 10% erythrocyte hemolysis, an effect attributed to residual unbound pegylated surfactant within the nanoparticle formulation [49]. These findings underscore the necessity for rigorous, systematic biocompatibility assessments of CPNs to comprehensively delineate their safety profiles within biologically relevant systems.

The lack of significant alterations in body weight, organ weights, hematological parameters, or serum biochemistry (ALT, AST, BUN, Cr) following single or repeated administrations indicates systemic biocompatibility. The transient elevation in fibrinogen levels at day 3 post-administration likely reflects a mild, self-limiting inflammatory response, consistent with the innate immune system’s recognition of foreign particles [50,51]. However, the return to normal values of this marker by day 14 suggests adaptive tolerance, a phenomenon observed with stealth nanoparticles engineered to evade immune detection. These findings contrast with reports on certain polymeric or metal-oxide NPs, which induce persistent inflammation or organ dysfunction at comparable doses [52,53].

Histopathological analyses further corroborated the safety profile, with no evidence of necrosis, fibrosis, or inflammatory infiltrates in major organs. The mild hydropic degeneration observed in hepatocytes at 30 days post-administration warrants attention, as it may signal early metabolic stress due to CPN accumulation in the liver. While this finding did not correlate with elevated liver enzymes or functional impairment, it highlights the need for chronic toxicity studies to exclude long-term hepatotoxicity.

Our findings align with the study by Wu et al. demonstrating that CPNs incorporating F8BT and poly(9,9-dioctylfluorene)-co-(4,7-di-2-thienyl-2,1,3-benzothiadiazole) (PF-5DTBT) exhibit exceptional biocompatibility and negligible reproductive toxicity in pregnant murine models, even at cumulative doses of 87.5–175.0 μg per mouse [54]. In contrast to cadmium-containing quantum dots (Cd-QDs)—which readily traverse the placental barrier, leach toxic heavy metals, and induce fetal mortality or developmental anomalies—CPNs displayed minimal placental transfer and no detectable adverse effects on maternal physiology, fetal development, or placental integrity. Crucially, the stable polymer architecture of CPNs mitigates risks of bioaccumulation-associated toxicity. This conclusion is substantiated by histopathological analyses revealing unaltered liver and spleen morphology, alongside unchanged serum biomarkers of hepatic function (AST, ALT, ALP), despite significant hepatic reticuloendothelial uptake [54]. Furthermore, the inert polymer backbone of CPNs circumvents the oxidative stress and inflammatory cascades typically triggered by inorganic NPs [55]. These collective results underscore the potential of CPNs as safer, biologically inert alternatives to conventional inorganic NPs for biomedical applications, particularly in contexts requiring stringent safety profiles, such as prenatal diagnostics or therapeutics.

The biodistribution pattern of CPNs, marked by hepatic and splenic accumulation, is characteristic of NPs cleared via the MPS [7,56]. The rapid blood clearance (within 24 h) and minimal renal retention suggest that CPNs are primarily metabolized through hepatobiliary pathways, reducing the risk of nephrotoxicity. This aligns with studies on similarly sized polymeric NPs, though surface modifications (e.g., PEGylation) could further modulate pharmacokinetics to enhance tumor targeting and reduce MPS uptake [57,58]. However, hepatic accumulation underscores the importance of long-term studies to evaluate potential metabolic stress or delayed hepatotoxicity.

While this study provides robust evidence of short-term safety, some limitations must be acknowledged. First, although the tested doses (≤1 mg/kg) were selected based on prior efficacy studies demonstrating therapeutic activity at lower doses (e.g., 0.3 mg/kg) in tumor-bearing models [33], dose escalation may still be necessary to achieve optimal therapeutic levels in complex clinical scenarios. However, the repeated-dose regimen (every 48 h over 28 days) enabled evaluation of significantly higher cumulative exposure (14 total doses), simulating a prolonged treatment schedule. This approach reflects an effort to infer potential chronic effects from a subacute model, though long-term studies remain critical. Second, the 30-day observation window precludes conclusions about chronic toxicity, which is critical for nanomaterials with potential organ accumulation. Future work should prioritize long-term studies, interspecies comparisons (e.g., in larger mammals). Additionally, functionalizing CPNs with tumor-targeting ligands could mitigate off-target accumulation and enhance therapeutic specificity. With continued interdisciplinary collaboration to refine biocompatibility, optimize dosing regimens, and elucidate long-term biodynamics, these porphyrin-doped CPNs hold immense promise to advance PDT into safer, more precise clinical paradigms, ultimately improving outcomes for patients with refractory malignancies.

The observed toxicity profiles primarily reflect inherent dark toxicity (baseline cytotoxicity independent of light activation), as the experimental design did not incorporate controlled irradiation at PS-specific wavelengths (λ ≈ 460 nm, Figure S4). While ambient light exposure during animal handling and housing could theoretically introduce photodynamic effects, such contributions are presumed to be negligible, as the maintenance cages were not positioned under direct light sources, minimizing unintentional PS activation, and no clinical signs of phototoxicity (e.g., erythema, ocular irritation, or tissue damage in light-exposed regions such as ears, paws, or skin) were observed in the animals. Nevertheless, the lack of systematic light exposure controls (e.g., shielded dark cohorts or irradiance measurements) precludes definitive exclusion of ambient light-mediated effects. Consequently, the reported toxicity data should be interpreted as encompassing both dark toxicity and potential, albeit undetected, photodynamic contributions of ambient light exposure. To further elucidate these mechanisms, subsequent work will include targeted assessments of CPN phototoxicity under controlled light exposure, enabling precise differentiation between dark and photoactivated cytotoxic effects.

Moreover, interspecies comparisons will allow for a more accurate characterization of the biodynamics of CPNs, including parameters such as clearance rate, hepatic metabolism, renal excretion, and tissue persistence, which are all critical aspects for designing safe and effective treatment protocols for human application. Prolonged post-treatment follow-up in these studies will also be essential to detect potential cumulative or late-onset effects that could compromise the long-term safety of NP-based therapies.

A key strategic direction in the evolution of these therapeutic platforms will be the functionalization of CPNs through the incorporation of high-affinity ligands targeting specific tumor biomarkers. This approach could not only enhance selective accumulation in tumor tissue but also minimize uptake in non-target organs such as the liver, spleen, and lungs, thereby reducing systemic toxicity and increasing the therapeutic index.

Additionally, the regulatory challenges inherent in the use of nanomaterials in hu-man medicine must not be underestimated. It will be essential to establish quality standards, reproducibility metrics, and physicochemical characterization criteria to ensure batch-to-batch consistency. Carefully designed clinical trials will be necessary to assess not only efficacy but also short- and long-term safety, including immunological and genotoxic parameters.

## 5. Conclusions

In summary, our study establishes a foundational safety profile for platinum porphyrin–doped F8BT CPNs, offering critical insights into their biocompatibility, biodistribution, and toxicity profile, which are essential steps toward their clinical translation for PDT application. Key findings include the excellent colloidal stability of CPNs in 5% dextrose, a clinically relevant vehicle, with minimal aggregation over time, ensuring suitability for parenteral administration. Notably, CPNs exhibited no hemolytic activity at concentrations up to 50 mg/L, confirming their blood compatibility and reducing risks of anemia or thrombotic complications. Single- and repeated-dose toxicity studies in BALB/c mice revealed no significant alterations in body weight, organ weights, hematological parameters (except transient fibrinogen elevation at day 3), or serum biochemistry (ALT, AST, BUN, Cr), underscoring systemic biocompatibility. Histopathological analysis further corroborated safety, with preserved tissue architecture in major organs and only mild hepatocyte vacuolation observed at 30 days, likely attributable to hepatic accumulation rather than overt toxicity. Biodistribution studies highlighted rapid blood clearance (within 24 h) and predominant hepatosplenic uptake, suggesting elimination via the mononuclear phagocyte system rather than renal excretion—a favorable profile for minimizing nephrotoxicity risks.

The significance of this work lies in its rigorous evaluation of CPNs under both acute and subacute exposure regimens, addressing a critical gap in the preclinical characterization of CP-based PSs. By demonstrating minimal toxicity and favorable pharmacokinetics, this study positions CPNs as promising candidates for clinical PDT, particularly for aggressive malignancies requiring repeated therapeutic interventions. Furthermore, the integration of colloidal stability, hemocompatibility, and in vivo safety data provides a robust foundation for regulatory considerations.

Future research should prioritize chronic toxicity studies to evaluate long-term organ retention and metabolic impacts, particularly given the observed hepatocyte vacuolation. Investigations in larger animal models (e.g., non-human primates) and tumor-bearing cohorts will further validate efficacy and safety in clinically relevant scenarios. Surface modifications, such as tumor-targeting ligands, could enhance circulation time and reduce off-target accumulation, while mechanistic studies elucidating CPN elimination pathways and immune interactions will refine their therapeutic utility. By addressing these gaps, CPNs could advance as a versatile platform for image-guided therapies, bridging the divide between nanomaterial innovation and clinical application.

## Figures and Tables

**Table 1:** The mean hydrodynamic diameter and PDI of CPN-PSPEG-PtOEP at 150 mg/L in different parenteral solutions used in this study (mean, *n* = 3).

Time (h)	Solution	d_h_ (nm)	PDI
1	water	29.0	0.25
0	0.9% NaCl	33.7	0.31
0.5	0.9% NaCl	35.0	0.29
1	0.9% NaCl	33.3	0.30
0	5% dextrose	24.9	0.34
0.5	5% dextrose	27.2	0.34
1	5% dextrose	28.2	0.34
0	PBS 1X	31.7	0.33
0.5	PBS 1X	35.3	0.35
1	PBS 1X	37.2	0.35

**Table 2:** The mean hydrodynamic diameter and PDI of CPN-PSMA-PtOEP at 150 mg/L in different parenteral solutions used in this study (mean, *n* = 3).

Time (h)	Solution	Mean Diameter (nm)	PDI
1	water	18.0	0.24
0	0.9% NaCl	23.4	0.32
0.5	0.9% NaCl	23.5	0.33
1	0.9% NaCl	29.1	0.38
0	5% dextrose	18.0	0.30
0.5	5% dextrose	14.9	0.40
1	5% dextrose	16.0	0.45
0	PBS 1X	20.6	0.38
0.5	PBS 1X	26.2	0.39
1	PBS 1X	30.0	0.45

## Data Availability

The original contributions presented in this study are included in the article/Supplementary Material. Further inquiries can be directed to the corresponding author.

## Supplementary Materials

The following supporting information can be downloaded at: https://www.mdpi.com/article/10.3390/pharmaceutics17050593/s1, Figure S1. Dynamic light scattering (DLS) analysis of CPNs. The hydrodynamic diameter distribution of CPN-PSPEG-PtOEP (blue) and CPN-PSMA-PtOEP (orange) is shown. The data indicate differences in nanoparticle size distribution, with CPN-PSPEG-PtOEP exhibiting a larger hydrodynamic diameter compared to CPN-PSMA-PtOEP. Figure S2. Body weight progression and organ weight distribution in experimental groups. (A) Line graph depicting body weight changes over time in the CPN-PSPEG-PtOEP and CPN-PSMA-PtOEP groups compared to the control group. (B,C) Organ weight comparison in the CPN-PSPEG-PtOEP (B) and CPN-PSMA-PtOEP (C) groups at different time points following a single-dose administration of nanoparticles. ns = no statistically significant differences. Figure S3. Biochemical analysis of organ function following CPN-PSPEG-PtOEP single-dose administration at different times and compared to control group. Figure S4. (A) Spectra characterization of CPNs stabilized with PSMA in water (red), PtOEP in deoxygenated THF (black), and CPN-PSMA-PtOEP in water (blue). (B) Emission spectra of CPN-PSMA in water; emission spectra were collected with excitation at 455 nm for F8BT (black).

## Abbreviations

ALT	Alanine Aminotransferase
ANOVA	Analysis of Variance
AST	Aspartate Aminotransferase
BUN	Blood Urea Nitrogen
CPNs	Conjugated Polymer Nanoparticles
CPs	Conjugated Polymers
Cr	Creatinine
DLS	Dynamic Light Scattering
EDTA	Ethylenediaminetetraacetic Acid
F8BT	Poly(9,9-dioctylfluorene-alt-benzothiadiazole)
Hb	Hemoglobin
H&E	Hematoxylin and Eosin
HTC	Hematocrit
ICP-MS	Inductively Coupled Plasma Mass Spectrometry
i.p.	Intraperitoneal
i.v.	Intravenous
MCV	Mean Corpuscular Volume
MCH	Mean Corpuscular Hemoglobin
MCHC	Mean Corpuscular Hemoglobin Concentration
MPS	Mononuclear Phagocyte System
N_3_-PEG-NH_2_	poly(ethylene glycol) derivative
NIH	National Institutes of Health
NPs	Nanoparticles
PBS	Phosphate-Buffered Saline
PDI	Polydispersity Index
PDT	Photodynamic Therapy
PEG	Polyethylene Glycol
PEGylation	Polyethylene Glycol Modification
PS	Photosensitizer
PSMA	Poly(styrene-co-maleic anhydride)
PFVBT	Poly[9,9-bis(N-but-3′-ynylN,N-dimethylaminohexyl)-fluorenyldivinylene-alt-4,7-(2′,1′,3′,-benzothiadiazole)dibromide]
PtOEP	Platinum(II) Octaethylporphyrin
RBC	Red Blood Cells
ROS	Reactive Oxygen Species
THF	Tetrahydrofuran
