# Supplementary material for: Preclinical Toxicological Characterization of Porphyrin-Doped Conjugated Polymer Nanoparticles for Photodynamic Therapy
Source: Pharmaceutics. 2025 May 1;17(5):593. doi: 10.3390/pharmaceutics17050593 (PMC12114993; doi:10.3390/pharmaceutics17050593)
Supplement: Supplementary file 1 [file pharmaceutics-17-00593-s001.zip › pharmaceutics-3604696-supplementary.pdf]

Supporting Information

Preclinical Toxicological Characterization of Porphyrin-Doped Conjugated Polymer Nanoparticles for Photodynamic Therapy

Matías Daniel Caverzan, [a] Ana Belen Morales Vasconsuelo, [b] Laura Cerchia, [c] Rodrigo Emiliano Palacios, [a] Carlos Alberto Chesta [a] and Luis Exequiel Ibarra [*b]

[a]. Instituto de Investigaciones en Tecnologías Energéticas y Materiales Avanzados (IITEMA), Universidad Nacional de Río Cuarto (UNRC) y Consejo Nacional de Investigaciones Científicas y Técnicas (CONICET), Río Cuarto X5800BIA, Argentina.

[b]. Instituto de Biotecnología Ambiental y Salud (INBIAS), UNRC y CONICET; Rio Cuarto X5800BIA, Argentina.

[c]. Institute of Endotypes in Oncology, Metabolism and Immunology "Gaetano Salvatore", National Research Council, 80131, Naples, Italy.

E-mail: libarra@exa.unrc.edu.ar

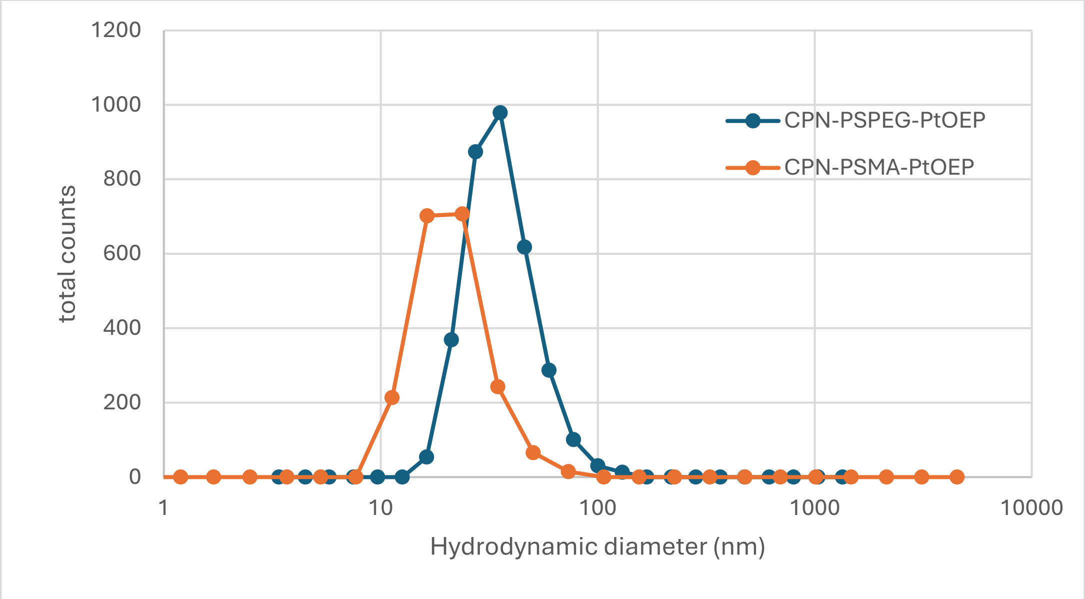

Figure S1. Dynamic Light Scattering (DLS) analysis of CPNs. The hydrodynamic diameter distribution of CPN-PSPEG-PtOEP (blue) and CPN-PSMA-PtOEP (orange) is shown. The data indicate differences in nanoparticle size distribution, with CPN-PSPEG-PtOEP exhibiting a larger hydrodynamic diameter compared to CPN-PSMA-PtOEP.

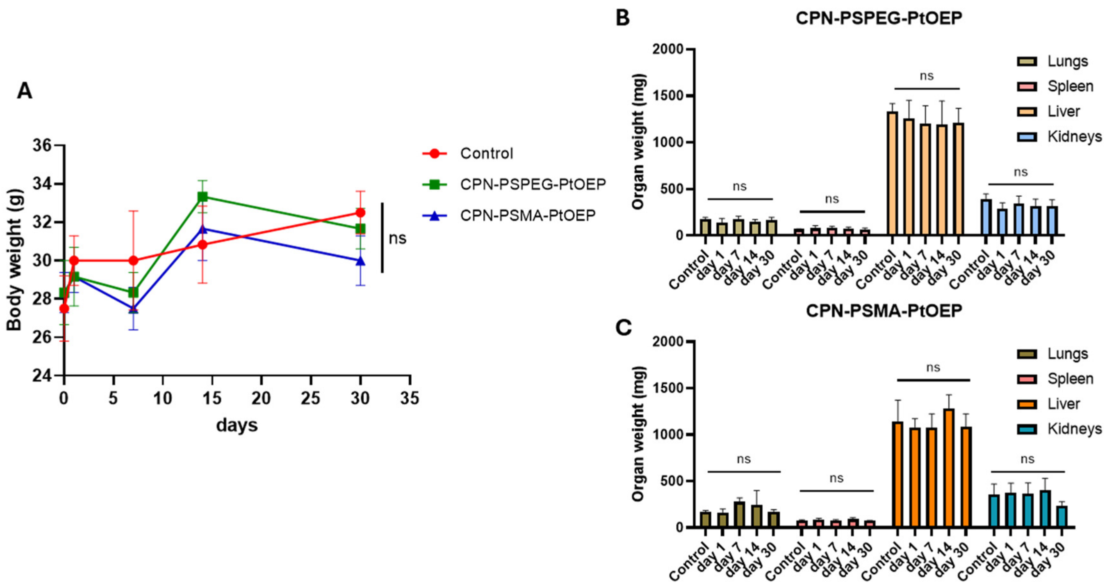

Figure S2. Body weight progression and organ weight distribution in experimental groups. (A) Line graph depicting body weight changes over time in the CPN-PSPEG-PtOEP and CPN-PSMA-PtOEP groups compared to the control group. (B-C) Organ weight comparison in the CPN-PSPEG-PtOEP (B) and CPN-PSMA-PtOEP (C) groups at different time points following a single-dose administration of nanoparticles. Ns = no statistically significant differences.

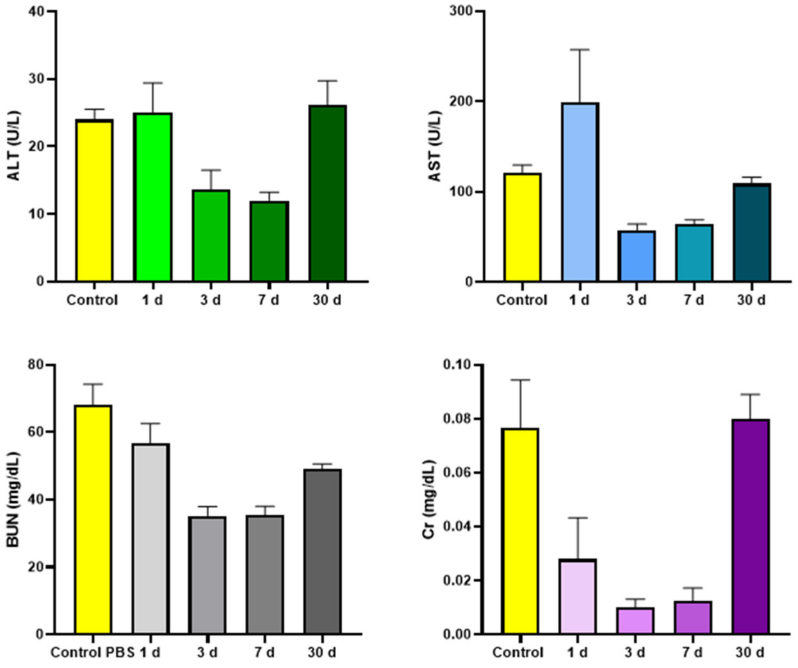

Figure S3. Biochemical analysis of organ function following CPN-PSPEG-PtOEP single-dose administration at different times and compared to control group.

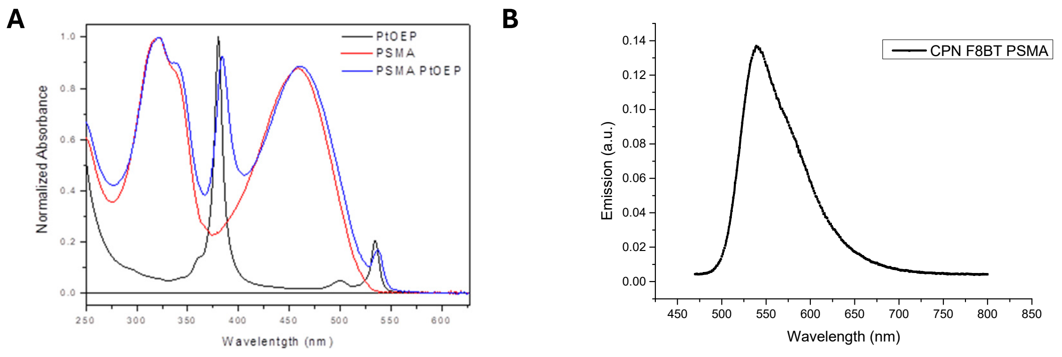

Figure S4. A. Spectra characterization of CPNs stabilized with PSMA in water (red), PtOEP in deoxygenated THF (black) and CPN-PSMA-PtOEP in water (blue). B. Emission spectra of CPN-PSMA in water; emission spectra were collected with excitation at 455 nm for F8BT (black).
